# Staying Together No Matter What: Becoming Young Parents on the Streets of Vancouver

**DOI:** 10.1007/s11013-022-09813-1

**Published:** 2023-01-24

**Authors:** Danya Fast, Reith Charlesworth, Madison Thulien, Andrea Krüsi, Jane Buxton, Sarah West, Corrina Chase, Daniel Manson

**Affiliations:** 1 Department of Medicine, Division of Social Medicine, University of British Columbia, Vancouver, Canada; 2 British Columbia Centre on Substance Use, Vancouver, Canada; 3 Centre for Gender & Sexual Health Equity, Vancouver, Canada; 4 School of Population and Public Health, University of British Columbia, Vancouver, Canada; 5 Indigenous Youth Researcher, Treatment Trajectories Study, British Columbia Centre on Substance Use, Vancouver, Canada; 6 First Nations Health Authority, Vancouver, Canada; 7 Métis, Vancouver, Canada

**Keywords:** Youth, Homelessness, Substance use, Pregnancy, Parenting

## Abstract

Among young people who use drugs in the context of entrenched poverty and homelessness, pregnancy is often viewed as an event that can meaningfully change the trajectory of their lives. However, youth’s desires and decision-making do not always align with the perspectives of various professionals and systems regarding how best to intervene during pregnancies and early parenting. Drawing on longitudinal interviews and fieldwork with young people in Vancouver, Canada, we explore how their romantic relationships powerfully shaped understandings of what was right and wrong and which actions to take during pregnancy and early parenting, and how these moral worlds frequently clashed with the imperatives of healthcare, criminal justice, and child protection systems. We demonstrate how a disjuncture between youth’s desires, decision-making and moralities, and the systems that are intended to help them, can further entrench young people in cycles of loss, defeat, and harm. These cycles are powerfully racialized for young Indigenous people in our context.

## Introduction

Young people who use drugs in the context of entrenched poverty and unstable housing and homelessness often view pregnancy as an event that can meaningfully alter the trajectories of their lives (Auerswald and Eyre 2002; Comfort et al. 2015). Many envision pregnancy as a time when they should reduce or eliminate substance use, secure more stable housing and steadier access to income, and seek out and solidify crucial forms of support (Begun et al. 2019; Knight 2015). In this way, youth’s perspectives often seem to converge with those of the various professionals in their lives, who also identify pregnancy as a strategic “window of opportunity” to make these kinds of significant changes (Smid, Bourgois, and Auerswald 2010). Despite this convergence, desired changes in young people’s lives are not often realized. Instead, pregnancy and early parenting can catalyze numerous harms for vulnerable young parents, including child apprehension, periods of mental health crisis, drug use relapse, binging and overdose, street-based homelessness, and disengagement from services (Begun et al. 2019; Cupers 2018; Kenny, Barrington, and Green 2015; Smid, Bourgois, and Auerswald 2010; Thumath et al. 2021). These harms are particularly alarming in the context of the current North American overdose crisis driven by the proliferation of illicitly manufactured fentanyl and related analogues in illicit drug supplies. In 2016, there were over 42,000 opioid overdose deaths in the USA (Gladden, Martinez, and Seth 2016). Since that year, over 1600 young people under the age of 30 have lost their lives to overdose in the Canadian province of British Columbia (British Columbia Coroners Service 2021).

Drawing on longitudinal qualitative and ethnographic research with young people in Greater Vancouver, in this paper we describe how their desires and decision-making surrounding pregnancy and early parenting did not always align with the perspectives of various professionals and systems regarding how best to intervene. In particular, we examine how youth’s romantic relationships shaped what they believed was right and wrong in particular moments, and how they ultimately navigated the demands of child protection, criminal justice, substance use treatment, and healthcare systems across time. We demonstrate that interventions which separated young couples were often experienced by youth as destabilizing the already fragile material, emotional, and moral worlds they had built for themselves. They undermined the very thing—their romantic relationships—that they viewed as essential to successfully navigating pregnancies, creating families, and building different kinds of lives. A disjuncture between youth’s desires, decision-making and moralities, and the systems that are intended to help them, can further entrench young people in cycles of loss, defeat, and harm that may include death, particularly in the context of the current crisis (Thumath et al. 2021). These cycles are powerfully racialized for young Indigenous people in our context.

Previous work by medical anthropologists and others has deftly illustrated how the reciprocal sharing of resources, drugs, and emotional support incurs mutual debts that equip individuals to better navigate the everyday emergencies of entrenched poverty, unstable housing, and homelessness (Bourgois and Schonberg 2009; Karandinos et al. 2014; Proudfoot 2017). While the professionals in young people’s lives often focus on the harms generated by their romantic relationships, research focused on youth has demonstrated that romantic relationships formed in these contexts are often crucial sources of material and emotional support, as well as sources of pain, violence, and instability (Begun et al. 2019; Cronley and Evans 2017; Joly and Connolly 2018; McCarthy and Casey 2008; Smid, Bourgois, and Auerswald 2010).

However, understandings of romantic relationships as either positive or negative, or even as positive *and* negative influences on young people’s trajectories do not fully account for the ways that these unions powerfully shape outcomes of pregnancy and early parenting in our and other similar settings. Instead, we follow anthropologist Jarrett Zigon (2013) in suggesting that romantic love, and by extension, relationships, are better conceptualized as “moral assemblages”^1^ composed of multiple, shifting, and at times contradictory understandings, experiences and desires, as well as embodied dispositions shaped by individual and collective histories. As moral assemblages, romantic relationships open up particular ethical ways of being in the world (Lenhard 2017). Importantly, they come to issue “unavoidable demands” (Zigon 2013: 201) for particular kinds of action in particular moments, including moments of decision-making surrounding how to navigate pregnancy and early parenting in a context of entrenched poverty, unstable housing and homelessness, and substance use. Viewed through this lens, we are able to better understand moments when young people’s decision-making clashes with, and is powerfully undermined by, the mandates of healthcare, criminal justice and child protection systems, even when there are shared goals.

In our setting, the histories that come to shape romantic relationships as moral assemblages frequently include experiences of chronic residential and caregiver instability and loss of family and community that stretch back across young people’s own childhoods and across generations (Turpel-Lafond 2020). These histories also include repeated negative interactions with healthcare, criminal justice and child protection professionals, services, and systems (ibid.; Smid, Bourgois, and Auerswald 2010). For the Indigenous young people who have participated in our research, these experiences across time and place must be understood as forms of ongoing and intergenerational colonial violence. There are currently three times more Indigenous children in government care than at the height of the residential school era (Blackstock, Bamblett, and Black 2020), and the rate of child welfare investigations into Indigenous families is between 4.2 and 8 times higher than for non-Indigenous families (Truth and Reconciliation Commission of Canada 2015), leading many to argue that the child protection system in fact constitutes “a new era of Indigenous child apprehension” in Canada (Pearce et al. 2019:53). Following Unangax̂ scholar Eve Tuck (2009), in this paper we want to both acknowledge the disproportionate harms experienced by Indigenous young people at the hands of contemporary systems and services that continue to be rife with racism, and move beyond these kinds of damage-centered narratives, to foreground young Indigenous people’s desires for love, family, self-determination, and different kinds of futures.

## Methods

This paper draws on 14 months of qualitative and ethnographic research with 16 youth conducted by the second author (Reith), a white settler, cisgender female researcher in her 30s. It also draws on a program of anthropological research conducted by the first author (also a white settler, cisgender female researcher in her thirties) since 2008 that has included hundreds of young people who use drugs in Greater Vancouver. Here, we focus on the experiences of a small number of individuals, utilizing critical phenomenology to develop granular, nuanced descriptions of these young people’s everyday lived experiences across time, while situating them within social, political, historical, and institutional contexts that are themselves shaped by larger matrices of power and political economy (Garcia 2010). In doing so, we build on previous anthropological research that demonstrates how carefully tracing individual patterns of experience can be productive for making and unmaking what is known about a collectivity and collective problems (Biehl 2005; Garcia 2010; Meyers 2013).

The 16 youth participants were recruited from the At-Risk Youth Study (ARYS), an ongoing prospective cohort study of over 1000 young people who use criminalized drugs in Greater Vancouver that began in 2005 (Wood et al. 2006). These participants ranged from 21 to 29 years of age at first interview, with a median age of 25. Some participants were navigating pregnancies while participating in the study, while others reflected on past pregnancies and their experiences as young parents in subsequent years. Participants were between the ages of 16 and 26 when they first learned about a pregnancy, with a median age of 22. Eight participants self-identified as Indigenous, seven participants self-identified as white, and one participant did not want to disclose their ethnicity. Four participants were in romantic relationships with partners who were also enrolled in the study (for a total of 8 youth). Six participants were in romantic relationships at the time of the study, but their partners were not enrolled in the study. The remaining two participants were not in romantic relationships for the duration of the study.

Consistent with a critical phenomenological approach, the primary form of data collection for this study was longitudinal in-depth, semi-structured interviews (Guba and Lincoln 1994). An interview guide was developed through engagement with previous literature and conversations with our team’s Youth Health Advisory Council (YHAC). The YHAC is a group of 10 youth with lived and living experience with substance use in the context of unstable housing and homelessness, including youth who have experienced pregnancy and parenting in this context.

First-time interviews occurred at the ARYS field office, where participants also provided their written informed consent to participate in the study. Consistent with a more ethnographic approach, follow-up interviews were conducted with participants in the settings of their everyday lives whenever possible. Situating these conversations in the day-to-day routines of young people often prompted new lines of questioning that did not come up during the more formal interviews at the ARYS office. In total, 37 interviews were conducted with the 16 youth participants. Interviews with participants occurred over approximately 1 year, beginning in May 2019 with follow-up interviews continuing until July 2020.

Interviews with youth were initially designed to elicit broad discussions of their experiences navigating pregnancies and early parenting, with a focus on how these experiences intersected with substance use and substance use treatment trajectories. Informed by a constructivist understanding of knowledge production, participants largely guided our conversations according to their own priorities and ways of understanding their circumstances (Thorne 2000). As interviews progressed, questions increasingly focused on participants’ past and current decision-making related to pregnancy and parenting in the context of romantic relationships and other kinds of social support. The interview questions also became more focused on youth’s interactions with healthcare, criminal justice, and child protection systems. Interviews lasted approximately 1 hour and participants received a $30 honorarium for their time and expertise. Each participant was offered the opportunity to do follow-up interviews every one to three months. Follow-up interviews were completed with 11 participants, including three of the four enrolled couples (for a total of 6 youth), whom Reith typically interviewed as a pair. All participants who completed follow-up interviews completed more than one. During follow-up interviews, participants reflected on how their circumstances had changed (or not) during the time period between conversations. Follow-up interviews were also an opportunity to discuss emerging interpretations of the data with participants, and more explicitly involve them in the co-creation of study findings.

In addition to in situ interviews, fieldwork activities also included spending time with young people in the places that they regularly frequented, including parks, coffee shops, and residences. These outings typically lasted 1 to 2 hours, and participants were compensated for their time with a $30 honorarium. Reith also maintained more regular (at least monthly) contact with 4 young people through phone calls, text messages, emails, and Facebook Messenger. Fieldwork outings and phone calls were documented through written fieldnotes, which described contextual factors that were not necessarily captured in audio recordings.

All interviews were audio-recorded and transcribed verbatim. Interview and fieldnote data were managed using *NVivo 12* software. After reading through the transcripts of early interviews and fieldnotes, the authors generated an initial coding framework that captured broad emergent themes (e.g., romantic relationships) and analytic categories (e.g., racism, gender). As interviews and fieldwork progressed, we revised these codes and created new ones (e.g., moral worlds) to reflect our evolving interpretations of themes and patterns in the data. These interpretations were also shaped by regular engagement with the YHAC during semi-monthly meetings across the study period. As is common in qualitative and ethnographic research, data collection and analyses occurred concurrently as the study progressed. This allowed us to rework and refine the coding framework based on subsequent interviews and fieldwork in an iterative and generative process.

Ethical approval for all study activities was obtained from the University of British Columbia’s Behavioural Research Ethics Board (H18-03256).

## Study Setting and Participants

The young people in this study had to simultaneously contend with the everyday emergencies of poverty and addiction alongside the challenges of pregnancy and early parenting, and the related demands of healthcare, criminal justice, and child protection systems. The everyday emergencies they faced included frequent evictions, unstable housing and homelessness, food insecurity, regular mental and physical health crises, and frequent interactions with law enforcement (Alexander, Bruun, and Koch 2018; Begun et al. 2019; Bourgois and Schonberg 2009).

Although there has been a scaling up of housing for low-income people who use drugs in Vancouver, including youth-dedicated government-subsidized supportive and temporary modular housing (Fast and Cunningham 2018), the majority of the young people in this study were sleeping outside with a romantic partner in downtown Vancouver at the time of the discovery of a pregnancy. Some had rooms in rundown, privately owned single room occupancy hotels (SROs), and a few had rooms in supportive or temporary modular housing buildings. As we will describe in more detail, experiences of street-based homelessness and unstable housing continued for many youth across the study period, because the supportive and temporary modular housing available to young people currently includes very few family housing options that would allow young couples to reside together with their child or children.

Vancouver is home to some of the most comprehensive and progressive programs for young people who use drugs in Canada, including community-based, residential, and in-patient hospital programs for pregnant and newly parenting individuals. These programs are generally informed by a harm reduction approach, and intended to keep infants and children with their parents whenever possible (BCCSU 2018; Hippola 2006; Poole 2000; Seaman 2004). They recognize the significance of safe and stable housing and financial stability in shaping outcomes for young pregnant and parenting individuals. However, grossly inadequate monthly income assistance payments, Vancouver’s exorbitant housing market, and a lack of government-subsidized housing for young families continue to present major challenges to supporting young people who are navigating pregnancy and parenting in the context of entrenched poverty and homelessness.

All study participants were engaged in some form of criminalized substance use across the study period, most commonly the daily use of fentanyl and/or crystal methamphetamine (meth). A number of participants also consumed alcohol daily. While some youth still referred to using “heroin,” or stated more generally that they used “down” (a slang term for illicit opioids), during the study period it was generally recognized that illicit opioids obtained in Vancouver’s street-based drug markets consisted primarily of or were heavily adulterated with illicitly manufactured fentanyl. It is for this reason that we use the term “fentanyl” throughout this paper.

Even as various systems and services are working towards becoming more culturally safe in British Columbia (First Nations Health Authority 2017), the racism that permeates healthcare, criminal justice, and child protection systems makes accessing and working within these systems difficult or impossible for many Indigenous young people, with devastating effects (Turpel-Lafond 2020). Eight of the 16 young people in this study self-identified as Indigenous. The intergenerational effects of governmental policies such as the Indian Act of 1876, the Indian Residential School system, and the Sixties and Millennium Scoops (throughout which thousands of First Nations, Métis, and Inuit children have been taken from their families and placed into foster care or put up for adoption) have systematically deprived Indigenous people of access to land, family, income, education, housing, healthcare, and food security—all of which contribute to disproportionate rates of substance use, poverty, and related inequities in the present (Interagency Coalition on AIDS and Development 2019; Firestone, Tyndall, and Fischer 2015; Martin and Walia 2019).

Most of the participants in this study—Indigenous, and non-Indigenous—shared that their own childhoods had been characterized by chronic poverty, caregiver and residential instability, and child protection services (CPS) involvement. And yet, almost all participants were excited to learn about a pregnancy, and saw it as an opportunity to dramatically change their lives and create a “real” family that was better than what they had experienced as children. Participants longed to avoid CPS involvement following the birth of their child. When these desires were not realized, it engendered a somewhat paradoxical sense of being utterly unsupported by and trapped within systems whose control extended not only across their own lives, but also across generations.

## Findings

### Staying Together No Matter What

Young people who were in romantic relationships described how their day-to-day lives largely revolved around these unions. Youth described spending “every moment” with their romantic partner: moving through the city, generating income, buying and using substances, and socializing and resting together. Youth almost always viewed their romantic partners as family. They frequently declared, both in person and on Facebook, that they were married, even if this was not the case legally. They wore matching rings, and graffitied the walls of their rooms and the places where they slept with fervent declarations of love and long-term commitment.

Pregnancy was described by both young men and young women as a happy, if also overwhelming, event that could stabilize oftentimes tumultuous romantic relationships and deepen a sense of romantic love and commitment between partners. With the exception of one couple, all participants who were in romantic relationships described envisioning becoming parents together and maintaining custody of their children when they learned about a pregnancy. Reith met 24-year-old Curtis and his girlfriend Farrah in November 2019 when they were about 3 months into a pregnancy. As Curtis described:

Honestly, the pregnancy has made us get closer, less fighting. We’ve seen the ultrasound, and it’s made me – it’s really bringing back love, intimacy, you know? It’s amazing. And **I’m just thinking about life in the future, and how it’s not going to be a letdown to myself, to the baby, to Farrah, to anybody.** It’s a miracle and a half. It’s motivation to change our lives.

Young people’s moral worlds were powerfully anchored by their romantic relationships, and they spent a great deal of time talking with Reith about what kind of behaviors were right and wrong in these contexts. Pregnancy was an event that seemed to ignite these moral worlds, deepening imperatives to behave in certain ways (e.g., be a “good” romantic partner and future parent). In May 2019, 23-year-old Dylan described his attachment to his girlfriend Amanda’s pregnancy 1 year prior, and his fierce commitment to being a good father:

Every time Amanda would be sleeping, I would always just stay up all night and talk to him [his unborn son]. And he would kick back and then **I would tell him that I was really, really excited for him to come, you know? To be born. And that I would be a really, really great father and everything.** And then he would always kick back. It was really great.

As “moral assemblages” (Zigon 2013) composed of multiple, shifting, and at times contradictory understandings, experiences, and desires, romantic relationships included gendered notions of how a young man should take care of and provide for his pregnant girlfriend/wife and future child. This is evident in how Dylan described taking care of Amanda during her pregnancy in the context of ongoing homelessness:

We were actually homeless for, like, a year while she was pregnant. We were sleeping outside. Since she was pregnant, I knew that it would be, like, a stupid idea to let her carry anything, so I carried all the bags and I made sure that she didn’t have to carry anything and that she was always eating, out of the both of us. You know? If only one of us had a chance to eat, I would make sure she was eating. **I would make sure she was okay, you know? I’m glad I was able to be there for her.**

Gendered notions of how a young man should support and provide for his pregnant girlfriend/wife intersected with embodied dispositions shaped by the demands of the everyday emergencies of poverty, homelessness, and addiction, to powerfully shape decision-making surrounding pregnancy and parenting. Both young men and young women often declared that a couple must “stay together no matter what” throughout pregnancy, childbirth, and early parenting to love, protect, and provide for each other. This seemed to be especially important to couples who had experienced chronic caregiver and residential instability and CPS involvement during their own childhoods, as Dylan and Amanda had.

A commitment to staying together no matter what often clashed with the imperatives of healthcare, criminal justice, and child protection systems, which frequently mandated a separation of young couples for periods of time during pregnancy and early parenting. For example, CPS social workers often dictate that one or both romantic partners attend residential treatment if they want to maintain custody of their child following birth. Due to the lack of residential treatment options that allow young couples to attend together, a decision to attend residential treatment was also a decision to separate for a period of time. Like the CPS social workers, the young couples who participated in this study often also envisioned pregnancy and early parenting as times to reduce and eliminate their substance use. Yet, when faced with the dilemma of whether or not to separate for a period of time, many young people decided that what was right for them was to stay together, even as professionals issued stern warnings that doing so would mean CPS involvement and most likely losing custody of their child. Youth expressed tremendous frustration and a painful sense of confusion regarding the ways that social workers, law enforcement, and other professionals endeavored to “break couples apart” and “did nothing to keep families together,” because from their perspective, this was fundamentally wrong.

For example, Dylan told Reith that when Amanda accessed a residential treatment program for pregnant women late in her pregnancy, the outreach workers there strongly encouraged him to access a different residential treatment program in anticipation of their child’s birth. But Dylan’s assessment of the situation was different:

**I didn’t want to leave her, because what if she needed me?** Before she was pregnant, we were homeless, and then while she was pregnant, we were homeless. And through those three years, she really couldn’t rely on anyone except me. And she knew that I was the only one that was there for her and would always be there for her, and **I was always willing to help her, you know, no matter what. And that was when our relationship was the best.**

Twenty-six-year-old Elijah described a thought process that was remarkably similar to Dylan’s. In this case, it was a restraining order that was preventing him from seeing his partner Kayley, who was 5 months pregnant at the time. Elijah’s parole officer (PO) told him that he must complete some form of substance use treatment before his court date in early December 2019 in order for the restraining order to be lifted. But he had decided not to go, and continued to see Kayley despite the restraining order:

It’s just that I would have been trapped inside for two weeks [in residential treatment] when **my girlfriend needs me right now.** It’s hard to do that when she’s pregnant and she needs me. Like, I’m pretty sure I’ll go back to treatment when she doesn’t. But **I’ve never left her side for three years, besides the three months I was in jail.**

In moments of dilemma, such as the decision of whether or not to go to residential treatment or abide by a restraining order, young people grappled with a “range of possibilities for morally being in the world and ethically working on oneself” (Zigon 2013: 202). As moral assemblages, romantic relationships issued certain kinds of “unavoidable demands” (ibid.: 201) such as the imperative to stay together no matter what. However, it is important to note that there were often other, competing demands at play, including those generated by the need to continually hustle for money, drugs, alcohol, food, and basic necessities in the context of chronic poverty and criminalization. For example, as Elijah acknowledged in the above quote, he insisted on not leaving Kayley’s side while simultaneously describing how he did leave her for 3 months while he was in jail. In February 2020, about a month after Kayley gave birth to their baby, Elijah was arrested again and spent several more days in jail, unable to contact Kayley while she cared for their newborn.

### Becoming Parents Under the Gaze of “The System”

All of the young people who participated in this study became parents under the “inspecting gaze” of CPS (Juhila et al. 2020: 5). This surveillance took the form of supervision orders issued by social workers that restricted contact between young people and their children and partner, and child apprehensions. Consistent with a large body of previous research (Kenny et al. 2021, 2015; Marsh and Leamon 2019; Thumath et al. 2021), the event of child apprehension was described by youth as abrupt and traumatic, usually leading to periods of mental health crisis, substance use relapses and binging, and the breakdown of romantic relationships. Alarmingly, no participants recalled being offered any mental health or other forms of support in the immediate aftermath of a child apprehension. Instead, youth framed their interactions with the CPS social workers who were in charge of their and their children’s files as overwhelmingly negative, painfully confusing, and, for the Indigenous young people in our study, shaped by discrimination against those who were, as one young Indigenous woman put it, “low income and Native.” Young couples consistently used language like “they are purposely sabotaging us” to describe their relationships with CPS social workers, who, as described above, frequently mandated periods of separation, thereby undermining the very thing that anchored young people’s fragile material, emotional, and moral worlds. As 27-year-old Katie and 29-year-old Mike described in October 2019, repeated encounters with a frenetic rotation of CPS social workers could leave young parents exhausted and feeling like they were “getting nowhere” (see also Fast 2021):

Reith: There’s so many [CPS social] workers I can’t keep them straight.

Katie: Yeah, like, both our kids have their own [CPS social] workers, and then we have our own [CPS] social worker—

Mike: We’ve had four [CPS] social workers. The first one got us to sign the CCO [continuing custody order, which legitimizes permanent foster care], and immediately after we signed the CCO, our kid was gone. And—

Katie: Then it was a new [CPS] social worker.

Mike: And then, **by the time we thought we were making progress on the things they were asking us to do, we got a new worker again.** Like, every time, we thought, “Hopefully this time we can move forward.” “Okay, she can’t be as bad as the last one.” **And we never got anywhere.**

Despite the fact that young people’s social workers changed so frequently, they were acutely aware that their files followed them from professional to professional and system to system. The content of these files often diverged dramatically from how youth envisioned themselves as romantic partners and parents. Twenty-one-year-old Liam was in a juvenile detention center when his child was born in 2014. Upon his release, Liam received a supervision order followed by a no-contact order that legally prohibited him from seeing his girlfriend Lisa and daughter.^2^ Liam continued to cycle in and out of juvenile detention centers; at least twice because he broke the conditions of the no-contact order by continuing to talk to Lisa. Lisa maintained custody of their child with the support of her parents, and eventually ended her relationship with Liam. In the summer of 2019, Liam voluntarily accessed a residential treatment program, citing his desire to “be a good father” as motivation to reduce his fentanyl use. A few months later, while he was living at the program’s second-stage recovery home, he told Reith over the phone:

In the Ministry’s defense [of implementing the supervision and no-contact orders], my file didn’t look very good, right? Like, I had a lengthy criminal record, you know, a history of addiction. I get where the fear comes from. But, I like to think that I’ve been trying and that I have a different personality than a lot of these people who are just not really giving a fuck. And for me, **it’s been something that’s been seriously important to me – that I know I have the potential to be a good father. It’s just, you know, it feels like a lot of the time I don’t really have the opportunity to prove that.** It’s tough when they [CPS social workers] don’t see the day-to-day stuff. They don’t see what you’re doing to make your life better. They only see you maybe once a week for an hour in the office when they ask you some questions.

Dylan was also under a supervision order immediately following his child’s birth in 2018, which prevented him from visiting Amanda or their child without professional supervision until he secured housing and employment. Without Amanda’s support, however, finding housing and a job seemed utterly impossible. Overwhelmed by the situation, he began avoiding meetings with his CPS social worker altogether. A year later, he identified the supervision order as the catalyst for Amanda ending their relationship:

When we had the social workers show up in our life, we were together and we were in love with each other and we wanted to stay with each other. **But then by the time the social workers left, they basically broke us apart, they basically made her hate me, and they basically made her not want to be with me.** It’s like, the whole supervision order broke us apart.

Mike similarly concluded that it was the no-contact order that he was under while Katie was attending a residential treatment program for pregnant women that ultimately caused their plans to “fall apart” and his own life to “go downhill.” It was at that time that he relapsed on fentanyl for 6 months.

Despite the various ways that young couples were separated from each other and their children, many continued to communicate with romantic partners and see their children secretly, risking further legal consequences. Liam went back to a juvenile detention center twice because he continued to talk to Lisa. Elijah secretly visited Kayley despite the fact that there was a restraining order in place (which Kayley told Reith she had been “forced” to sign so that Elijah could avoid jail time). Elijah believed that the legal mechanisms used to separate him from Kayley were shaped by systemic racism, describing how a sense of being surveilled, controlled, and criminalized left him feeling “almost broken inside.” In 2019, he had been on probation for almost the entirety of the previous 6 years:

The cops downtown, they don’t care, they just – sometimes **it seems like they’re racist. They just make life hard for certain types of people.** They picked on me and my brother a lot. They were always breaking in my room [at the SRO], searching my room and stuff.

As mentioned above, Elijah was told by his PO that the only way the restraining order would be lifted was if he attended a substance use treatment program.

### The Imperative of Substance Use Treatment

Upon learning about a pregnancy, young people began envisioning different kinds of futures that included stable housing, steadier employment or adequate income support, and, for many, a deepened connection with and commitment to their romantic partner. Youth acknowledged that reducing or eliminating intensive substance use was critical to realizing these futures, and most were open to the possibility of some form of substance use treatment. To this extent, their understandings of what was required to change the trajectories of their lives aligned with the CPS imperative that they engage in substance use treatment if they wanted to maintain or regain custody.

Yet, a painful sense of frustration and confusion often arose as youth attempted to make progress in the ways demanded by CPS social workers but were nonetheless continually denied custody of their children, as well as the forms of support—namely, stable family housing—that would allow them to “prove” that they could be good parents. For example, even after successfully completing a treatment program during her pregnancy and maintaining abstinence from substance use (demonstrated through regular urine toxicology screens), Katie was denied custody after the birth of her second child. Despite a deep sense of disappointment at not being granted custody, Katie maintained abstinence from all substance use, and she and Mike were able to move in with her own parents. When Reith met the couple in October 2019, they were attempting to begin the process of reunification with their two children. Eventually, in January 2020, Katie and Mike’s children’s files were transferred to a different child protection agency after Katie disclosed her Indigenous ancestry. Both Katie and Mike were optimistic about this transfer. They felt like the social worker from the new agency was “working *with* them” and wanted to help them regain custody of both of their children. However, the new worker mandated that both parents attend residential treatment before receiving housing support, despite the fact that Katie had maintained abstinence from substance use since the birth of her second child, and Mike had maintained a Suboxone (buprenorphine-naloxone) prescription for 9 months:

Reith: And so, is [the requirement to go to residential substance use treatment] basically just to check a box?

Katie: Well, no, that box is already checked. Like, I was clean for the year, going to different treatment centers, different places, and they won’t tell me when me and Mike can have our own place together, and, like, live our – live our life.

Mike: I’ve been waiting to get into [residential] treatment, just to get things going to get to the next step of being able to have custody of our kids and live independently. But, it’s almost like we’ve been hung out to dry. It’s like, okay, now that we’ve gotten clean, you want us to go back to [residential] treatment again for whatever reason, **and now we’re kind of left in limbo to, like, maybe fuck up before we can do any better. Like, we’re being left in that grey area between, like, you’ve done it, and you’re almost there.**

As Mike described, being “left in limbo to maybe fuck up” was a disastrous situation for many youth. The frustration, confusion, and exhaustion that was generated by continually attempting and then failing to meet the demands of CPS social workers and POs could lead to periods of mental health crisis and substance use relapses and binges. Some young people became increasingly resigned to the fact that they were “not getting anywhere,” as Mike put it, despite their efforts to “make progress” and demonstrate that they could be good parents. In this context, many made the decision not to attend residential treatment at all, or left residential treatment early to reconnect with their romantic partner and, in some cases, children.

### Cycles of Loss, Defeat, and Harm

Youth were navigating multiple, crushing forms of loss, including the loss of their child or children to “the system,” the loss of romantic partners, and the loss of family. They were also navigating various forms of defeat that, for some, transformed into hopelessness. Young people were most frequently offered support in the form of substance use treatment. However, all of those who participated in this study indicated that it was stable housing, employment, and income support that they most desperately needed to create a home for their child or children and family. Unfortunately, youth received little institutional support in accessing these. Moreover, the various mechanisms that separated young couples significantly destabilized their already fragile material, emotional, and moral worlds, frequently sending them into chaos. Recall that Dylan was told by his social worker that he had to secure housing and employment before he would be allowed to see Amanda and his child. In the absence of institutional and intimate support, Dylan experienced challenges in accessing even “low-barrier” work programs. Over the course of this study, Dylan was enrolled in at least three such programs, but either stopped attending or was kicked out. He explained:

Like, I barely had any, like, work experience, or any, like, qualifications, right? **It was harder for me to, like, basically, uh, get a grasp on the program, and get a grasp on what we were actually doing, right?**

Dylan also struggled with the complicated administrative processes involved in accessing low-barrier housing. Although he was living in supportive housing in May 2019, he was evicted in the fall of 2019 and became homeless again. He described attempting to get a room in another supportive housing facility, which required phoning regularly to maintain his place on the waitlist. However, Dylan could rarely make it past the automated menu options: “I don’t really get what they mean when – like, I try to call sometimes and then I only make it to the, like, menu, and that’s it. **I don’t even know where to go.**”

Dylan became increasingly despondent between the fall of 2019 and the spring of 2020, as he attempted to navigate poverty, homelessness, substance use, and the various expectations his social worker had set out in order for him to reconnect with Amanda and see his child. As he summarized in early 2020:

I asked Amanda the other day – I was like, “Do you honestly see a possibility of us ever being reunited as a family? Honestly?” **Like, that’s all I’ve been stressing about lately and I just want to know if I should keep going, trying to be motivated at this, or if I should just give up** because, like, I don’t know which way it’s going to go. I’m just feeling too overwhelmed. I’m feeling too exhausted, honestly. I just can’t seem to get out of bed for anything. I just hate it. **It’s just not what I expected I had to go through to be a parent. Like, all I ever wanted was to have a family.**

A year earlier, Dylan had told Reith how he longed to be “an actual dad” for his son, unlike the role his own father had played in his life. Now, it seemed to Dylan that this desire was hopelessly out of reach. Like all of the other young men in this study, Dylan was not aware of any mental health or other supports that he could access to help him manage the multiple losses and overwhelming sense of defeat that he was experiencing. Instead, he turned to what he referred to as “unhealthy coping outlets”—namely, the use of alcohol, meth, and fentanyl.

For Dylan and others, frustrations surrounding “getting nowhere” with CPS social workers, treatment programs, and housing and employment searches could make them feel like “giving up on” their plans for the future, and lead to periods of mental health crisis and harmful substance use relapses and binges. In January 2020, Katie and Mike felt like their CPS social workers were waiting for them to do just that:

Mike: I kind of feel like **our social workers are just waiting until something goes wrong, and for us to just throw up our hands and give up.** I’ve done that already where I was like, “Okay, well, I can’t do it, I give up,” and I went right back to my addiction.

Katie: It feels like our social worker was threatening us, like, **“You guys will always be in the system.”**

For the Indigenous young people who participated in this study, cycles of loss, defeat and harm at the hands of “the system” were deepened by the intergenerational trauma of settler colonialism and related forms of violence. Dylan, Farrah, Amanda, Elijah, Kayley, and Katie all identify as Indigenous. In 2018, shortly after the birth of his child, Dylan was attempting to manage his social worker’s expectation that he attend residential treatment while also dealing with the disappearance of his sister. At this time, there continued to be immense public pressure on the Canadian Government to investigate the overwhelming number of missing and murdered Indigenous women and girls that has long been an issue across the country, and a National Inquiry was underway to investigate the systemic causes of this violence (National Inquiry into Missing and Murdered Indigenous Women and Girls 2019a, National Inquiry into Missing and Murdered Indigenous Women and Girls 2019b). The stress of his sister’s disappearance led Dylan to leave treatment early. A short time later, Dylan learned that his sister had died by suicide. When the National Inquiry’s final report was published a year later, it concluded that the various forms of violence experienced by Indigenous women and girls, including suicide, are shaped by colonial oppression (National Inquiry into Murdered and Missing Indigenous Women and Girls 2019a, National Inquiry into Murdered and Missing Indigenous Women and Girls 2019b).

Dylan told Reith that he did not receive any mental health or other kinds of support to help him deal with this trauma. It is not possible for us to know whether this help was offered, or whether Dylan, like many others who participated in this study, felt like he didn’t know how to ask for help or where help might come from. After leaving treatment and learning about the death of his sister, Dylan became significantly re-entrenched in homelessness and intensive meth and fentanyl use.

## Discussion

This paper explores how pregnancy and early parenting shaped the trajectories of young people who were also navigating entrenched poverty, unstable housing and homelessness, and criminalized substance use. We mark the ways that young people’s desires for their romantic relationships, families, and futures circulated through experiences of pregnancy and early parenting, as well as how particular structural pressures and institutional arrangements often worked to cut them off from desired futures (Malins 2017). Our findings reveal a critical disjuncture between youth’s desires, decision-making and moralities, and interventions by healthcare, criminal justice, and child protection systems. For all of the youth who participated in this study, this disjuncture ultimately resulted in child custody loss.

While the number of young people enrolled in this study was small and findings should be generalized with caution, we observed that those who were in committed romantic relationships—a commitment that was often significantly deepened by the events of pregnancy and childbirth—seemed to have the most difficulty navigating the demands of these systems, even when all parties involved viewed the discovery of a pregnancy as a possible “turning point” for creating significant changes. At the time of writing, only two of the young women who participated in this study have been able to regain custody of their child or children. It is notable that these two young women were both not in romantic relationships while undergoing this process, and regained and maintained custody with significant support from a parent, which was not an option for most of the other young people in this study.

In our setting and others, romantic relationships are often held up by the various professionals in young people’s lives as sources of risk and harm, and as barriers to making changes such as reducing or eliminating substance use, securing more stable housing and steadier access to income, and seeking out and solidifying other forms of support (Joly and Connolly 2018; Smid, Bourgois, and Auerswald 2010). Indeed, in his ethnography of a couple undergoing substance use treatment at a rehabilitation center in Russia, Zigon (2013) describes how their romantic relationship deteriorated over time and they eventually began using heroin again, leading the staff at the center to use their story to warn others about how romantic love often interferes with the ability to remain abstinent from substance use. As Zigon notes, however, evaluating the impacts of the couple’s love solely in relation to the imperative to remain abstinent misses the other kinds of possibilities that are opened up by romantic love. In our context as in Zigon’s, romantic love and relationships can engender particular ethical ways of being in the world. They can produce a powerful sense of what is right and wrong and provide a guideline for action in particular moments. In our setting, we saw that these moral worlds were alighted by the events of pregnancy, childbirth, and early parenting, intensifying young people’s commitment to stay together no matter what even if that meant not going to residential treatment or leaving treatment early, and violating restraining and no-contact orders.

Interventions that separated young couples were often experienced by young people as destabilizing their already fragile material, emotional, and moral worlds. They undermined the very thing—their romantic relationships—that they viewed as a source of their power in the world. Systems and services were therefore not just experienced as fragmented and punitive (Comfort et al. 2015; López 2020) but as actively working against young couples even when, from their perspective, they were genuinely trying to “do the right thing.” This resulted in a painful sense of frustration, confusion, and defeat (or what could also be characterized as a dramatically reduced sense of self-efficacy), as well as cycles of loss, defeat, and harm. It could also result in re-traumatization when negative interactions with CPS in the present surfaced memories of young people’s own apprehensions during childhood. Indeed, the cycles of loss, defeat, and harm that young people were experiencing often extended both across their own lives as well as intergenerationally.

All participants identified having their children apprehended as another kind of “turning point”—this time, a profoundly negative one—after which many experienced drug use relapses and binges, and periods of mental health crisis and street-based homelessness (Kenny et al. 2021). These effects were most pronounced amongst the Indigenous young people who participated in this study. Dylan and another young woman in this study became homeless after losing access to their children, and Elijah described being “broken inside” after a restraining order prevented him from seeing his girlfriend and getting off probation. For Indigenous young people, intergenerational trauma could combine with negative experiences with CPS during their own childhoods and more recent experiences of racism in healthcare, criminal justice, and child welfare systems to engender a loss of hope for love and family. These experiences strongly discouraged them from accessing any much-needed services during pregnancy, early parenthood, and following a child apprehension (Turpel-Lafond 2020).

Negative experiences during pregnancy and early parenting produce intergenerational harms; they powerfully impact young parents, and their children, across the life course (Hertzman and Boyce 2010; Marmot and Bell 2012). Once placed in government care, children and youth face increased vulnerability to a multitude of health and social harms over time, including violence, unstable housing and homelessness, and substance use (Blackstock 2015; Martin and Walia 2019; Sherlock and Culbert 2017; Sinclair 2007). Yet, a recent cohort study of young, first-time pregnant women led by Catherine et al. (2019) demonstrates that, despite Canada’s high-income status and longstanding commitment to equity in healthcare and social service access, unacceptably high levels of disadvantage exist for many young mothers in British Columbia and Canada that, in turn, impact their children’s development. The authors conclude that “reaching these populations and addressing avoidable adversities during early pregnancy—thereby also increasing children’s life chances—is a societal imperative” (ibid.: 8).

During their pregnancies, Amanda, Katie, and two other young women in this study received valuable, albeit time-limited, support from specialized community-based and residential services for pregnant women—although it should be noted that this support did not ultimately allow them to maintain custody of their children after giving birth. Importantly, and consistent with previous research (Davies 2016; Devault et al. 2008; Mniszak et al. 2020; Smid, Bourgois, and Auerswald 2010), even these comprehensive and progressive services offered little to no support to the male partners of pregnant individuals, or to couples (Weaver 2013). Consequently, although the young men in this study were highly focused on becoming good fathers (it was usually framed as the most important role of their lives; see also Begun et al. 2019), they often experienced a sense of erasure as well as significant harms when their partners were drawn into women-only programs, leaving them isolated (see also Mniszak et al. 2019), and in some cases, without housing. At other times, young fathers were more forcibly excluded from their partner’s (via restraining orders) and children’s (via no-contact and supervision orders) lives. Under these kinds of pressures, their abilities to “do the right thing” often collapsed. The result of separations and exclusions for young fathers like Elijah, Liam, Mike, and Dylan were periods of substance use binging and relapse, mental health crisis, and street-based homelessness, as well as incarceration for violating conditions.

A significant lack of services dedicated to romantic couples and parents who are not mothers perpetuates assumptions that women are the natural bearers of responsibilities when it comes to pregnancy and parenting. It can place strain on co-parenting dynamics and a greater burden of responsibility on young women (Catherine et al. 2019). A lack of support for young fathers can become especially detrimental if the co-parenting relationship deteriorates, as it did for Dylan and Amanda. Gendered programming aimed at mothers also upholds heteronormative ideas of family that exclude gender diverse and queer youth. It should be noted here that much of the previous literature about young parents focuses on cisgender individuals in heterosexual relationships, and the experiences of trans, non-binary, Two Spirit, and queer youth are largely unaccounted for (Dietz 2020; Lowik and Knight 2019). This study contributes to the growing body of literature documenting the impacts of child apprehension and involuntary separation from romantic partners on young fathers (Bayley, Wallace, and Choudhry 2009; Davies 2016; Deslauriers et al. 2012; Devault et al. 2008; Mniszak et al. 2020). Unfortunately, it does not contribute to filling knowledge gaps regarding the experiences of gender diverse and queer youth experiencing pregnancy and parenting. Also missing from our study are the perspectives of child protection, criminal justice, substance use treatment, and healthcare providers, workers, and professionals on young people’s romantic relationships and evolving moral worlds, and how these complicate different kinds of institutional encounters across time. These are crucial areas for future research.

The findings of our study add to existing, urgent calls for public policy remedies that extend beyond healthcare interventions and encompass children and families (Catherine et al. 2019; Martin and Walia 2019). We identified a dire lack of low-barrier, supportive family housing for young people, including family housing for Indigenous youth. Only one couple in this study (Farrah and Curtis) were able to access supportive housing together, and it was intended to be temporary until the birth of their child. Our findings powerfully demonstrate that we must find ways to work *with* young couples who are navigating pregnancy and early parenting, even as these relationships are often highly tumultuous and can be the source of harms. Rather than making decisions for young people and couples, systems and services should focus on supporting their self-determination in relation to making meaningful changes in their lives. Several youth indicated that they would have attended residential treatment if they could have done so as a couple. In the absence of family programs that include or are aimed at young people, many of those in this study made the decision not to attend residential treatment at all, or left residential treatment early to reconnect with their romantic partner and, in some cases, children.

Finally, there is an unacceptable lack of mental health supports, including culturally safe programs, following the loss of child custody for young people navigating pregnancy and early parenthood in our context. It must also be noted that the young people in this study were also significantly harmed by the ongoing criminalization of substance use. When young parents such as Liam and Elijah were arrested, incarcerated, or remanded for substance use-related charges, it derailed their abilities to make desired changes in their lives. Recommendations from Indigenous activist Carol Muree Martin and activist Harsha Walia (2019) include expanding non-policing, Indigenous-led, and other community-driven approaches to responding to “criminal” behavior that is directly linked to substance use. Such approaches could provide essential opportunities to connect individuals such as Liam and Elijah with support rather than punishment.

Addressing the criminalization of substance use and homelessness, and designing family treatment and housing programs that are more congruent with diverse young people’s own understandings, experiences, desires and moralities, are crucial aspects of dismantling racism and other forms of structural oppression that systematically disadvantage Indigenous and other young people in our context. In collaborating with Indigenous peoples and organizations and others to develop and re-design equity-oriented family housing, treatment, and social interventions, we must center and respond to the ways that settler colonialism, systemic racism, and other oppressive forces influence the health and wellness of young people who are navigating pregnancy and early parenting in the context of entrenched poverty, homelessness and unstable housing, and criminalized substance use.

## Footnotes

1 Much recent work has utilized Deleuze and Guattarí’s (1987) concept of “assemblage” to explore the multiplicity and proliferation of relations that mediate substance use, policymaking, policing, recovery, and family making (Biehl and Locke 2010; Bøhling 2015; Dennis 2016; Dilkes-Frayne 2014; Duff 2014; McLeod 2017; Price-Robertson, Manderson, and Duff 2017; Price and Epp 2015; Søgaard, Houborg, and Pedersen 2017). This concept has been helpful for countering understandings that rely on static and linear causal models, instead demonstrating how particular configurations of people, places, practices, ideas, and policies are constantly being made, unmade, and remade through desires for things to be otherwise (Biehl and Locke 2010; McLeod 2017).

2 Unlike supervision orders, no-contact orders are issued by law enforcement and legally prohibit all contact between the individuals involved.
